# Beliefs About Children’s Memory and Child Investigative Interviewing Practices: A Survey in Dutch Child Protection Professionals from ‘Safe Home’

**DOI:** 10.3389/fpsyg.2020.546187

**Published:** 2020-09-25

**Authors:** Brenda Erens, Henry Otgaar, Lawrence Patihis, Corine de Ruiter

**Affiliations:** ^1^Clinical Psychological Science, Maastricht University, Maastricht, Netherlands; ^2^Leuven Institute of Criminology, Catholic University of Leuven, Leuven, Belgium; ^3^University of Portsmouth, Portsmouth, United Kingdom

**Keywords:** memory beliefs, child abuse investigation, child forensic interviewing, child protection, forensic interviewing

## Abstract

Knowledge of children’s memory and forensic interviewing skills are crucial in child abuse investigations. Safe Home is the Dutch hotline where both professionals and citizens can report concerns about child abuse or domestic violence. Professionals at Safe Home often serve as first responders to determine the need for a child abuse investigation, protective measures and/or further police investigation. In this study, child protection professionals (*N* = 158) employed at Safe Home (i.e., behavioral scientists, medical doctors, and social workers) completed an online survey on beliefs about memory functioning and forensic interviewing. In line with earlier studies, we expected to find a lack of knowledge about memory functioning among Safe Home workers. Furthermore, we expected limited use of forensic interviewing methods that have received empirical support. Indeed, we found many professionals endorsed beliefs not in line with current memory research, especially beliefs about repressed and recovered memories. Still, high percentages of professionals also reported memory beliefs related to false memory formation and suggestion that were in line with scientific evidence. Some professionals reported using interviewing methods for which there is no empirical validation. Because child protection professionals are often the first to interview children about allegations of abuse, the current findings identify a need for training in child forensic interviewing, including knowledge of human memory.

## Introduction

Child protection workers deal with complex cases of alleged child abuse on a daily basis. As part of their work, they interview children about alleged experiences of abuse. There are potential pitfalls in interviewing children as previous research has documented children’s susceptibility to suggestive interviewing techniques (e.g., Ceci et al., 2002). There are cases in which questionable interviewing techniques appear to have led to false memories of abuse in children (e.g., the McMartin case; Garven et al., 1998; see Otgaar et al., 2017, for a Dutch case). Use of evidence-informed child forensic interviewing methods, based on knowledge about children’s memory functioning (Cross and Hershkowitz, 2017) are therefore crucial. Professionals tasked with child abuse investigation need to know which types of interview questions are best at minimizing inaccurate memories of events, as well as producing detailed recall (Lamb et al., 2007).

Within the Dutch child protection system, the use of child interviewing protocols that are empirically validated is limited (Kinderombudsman, 2013). Articles that criticize the decision making process in the child protection system regularly appear in Dutch media (Huijer, 2014). For example, in their 2014 annual report, the Dutch Youth Inspectorate expressed concern regarding the lack of objectivity and fact finding within the child protection system (Inspectie Jeugdzorg, 2014). Recently, the Dutch Minister for Legal Protection announced a plan of improvement regarding fact finding in the child protection system (Dekker, 2018). The plan includes up to 20 strategies to improve child abuse investigations performed by different organizations.

### Child Protection Work at Safe Home

“Safe Home” (in Dutch: *Veilig Thuis*) is the Dutch hotline to be called in case of concerns regarding a possible case of child abuse or domestic violence. It was founded on January 1st, 2015. Safe Home employs social workers, behavioral scientists, and medical doctors. Currently, there are 26 regional Safe Home organizations in the Netherlands. These organizations provide advice on child abuse to professionals who work with children (e.g., school teachers, sports coaches) and to citizens, and they investigate allegations of child abuse and domestic violence. A citizen can file a report of possible abuse at Safe Home. During the first 6 months of 2017, Safe Home received 42,090 reports of alleged child abuse or domestic violence (Central Bureau of Statistics, 2017). In a child abuse investigation, Safe Home gathers information from a child interview and from collateral informants (mostly professionals, such as medical doctors, police, and school staff). This information is used to conduct a so-called safety assessment to determine if follow-up action is needed to ensure the child’s safety, both in the short and longer term. In case the investigation reveals serious abuse that could lead to criminal prosecution, Safe Home refers the case to the police as per government policy (Kwakman, 2017).

### Child Protection Work in Other Countries

It is important to realize that child protection services, such as Safe Home in the Netherlands, shares similarities with child protection systems in other countries. In the Netherlands, Safe Home often collaborates with the police and with local municipalities when investigating a report of alleged child abuse or domestic violence. The police are responsible for the criminal investigation, while the municipalities provide the necessary help or treatment to the families, provided by different mental health organizations. Safe Home consults with its relevant partners in the process of investigating and referring the case. This resembles, for example, the child protection system in England (led by the Department of Education), where local authorities, clinical commission groups and the police have a joint responsibility in protecting child welfare (Child protection system in England, 2019). They work together with other relevant agencies to ensure the effectiveness of work to protect and promote child welfare, such as making arrangements to identify and support children at risk of harm. Although Safe Home collaborates with its partners, it is the only organization in the Netherlands that provides advice in alleged child abuse cases and can start an investigation into the child’s welfare. For a comparison between Safe Home and child protective services in countries with similar economic and cultural characteristics (see Table 1).

**TABLE 1:** Comparison of child protection services in several countries.

Country	Organization(s)	Tasks	Collaborations
Netherlands	Safe Home (National hotline for child abuse and domestic violence) and the Council for Child Protection (can petition a supervision order at Court)	Give advice and investigate reports of alleged child abuse	Council for Child Protection, police, Public Prosecutor’s Office, municipalities
England	Local authorities work together with clinical commission groups and the police^a^	Investigating concerns and taking the necessary steps to protect the child	National Society for the Prevention of Cruelty to Children (NSPCC)
United States	The National Children’s Alliance (NCA) is the national association and accrediting body for a network of more than 850 Child Advocacy Centre’s (CACs) represented in different states^b^	CACs provide a coordinated, evidence-based response to children who have been abused in all 50 states	Law enforcement, prosecution, Child Protective Services, (mental) health institutions
Scotland	Child Protection Committees (CPCs) are responsible for multi-agency child protection policy, procedure, guidance and practice^c^	Investigating concerns and taking the necessary steps to protect the child	Within each local authority, CPCs work with local agencies, such as children’s social work, health services and the police
Sweden	Barnahus (children’s house)^d^	Derived from the CACs in the USA: represents a multi-professional approach to child victims of abuse with the double aim of facilitating the legal process and ensuring that the child receives necessary support and treatment.	Law enforcement, prosecution, Child Protective Services, (mental) health institutions

**^*a*^Child protection system in England. (2019, November). Retrieved from: https://learning.nspcc.org.uk/child-protection-system/england/^*b*^Child protection system in Scotland. (2019, July). Retrieved from: https://learning.nspcc.org.uk/child-protection-system/scotland/^*c*^How the CAC model works. (2019). Retrieved from: https://www.nationalchildrensalliance.org/cac-model/^*d*^Johansson, S., Stefansen, K., Bakketeig, E., and Kaldal, A. (2017). Implementing the Nordic Barnahus model: Characteristics and local adaptions. Collaborating Against Child Abuse, 1–31.**

Because many child abuse allegations do not include physical evidence (Euser et al., 2010), child investigative interviewing is a crucial element in child abuse investigations. However, child forensic interviewing is challenging and suggestive questioning could result in inaccurate statements (Ceci and Bruck, 1993; Lamb et al., 2011; Otgaar et al., 2018). In addition, several factors can make children reluctant to disclose abuse, such as feelings of shame and fear of the consequences after disclosure (McElvaney, 2015). When professionals interview children about events they have experienced, they need to tap into episodic memory of the child, i.e., memory for events/episodes. In the past few decades, a large body of research has uncovered ways in which interviewers can help or hinder episodic memory retrieval (e.g., Lindsay, 1990; Melinder, 2002; Goodman and Melinder, 2007), and professionals’ beliefs about memory functioning play a role in this (Melinder et al., 2004).

### Memory Beliefs of Professionals

Holding inaccurate beliefs regarding the functioning of memory could impact behavior of a Safe Home professional. For example, a professional who believes that certain psychological problems of a child (e.g., sleep problems) are linked to repressed memories of child sexual abuse, could become confident that a child is abused, even if the interview with the child uncovered limited factual evidence to support that conclusion. Previous research has examined memory beliefs among various professions and demonstrated a lack of knowledge concerning memory functioning among mental health clinicians (Gore-Felton et al., 2000), psychotherapists (Merckelbach and Wessel, 1998), and doctorate level clinical psychologists (Dammeyer et al., 1997). Recent studies revealed that many professionals hold beliefs that do not match current findings from memory research. For example, Ost et al. (2017) surveyed memory beliefs in Chartered Clinical Psychologists, unchartered therapists (hypnotherapists), and undergraduate psychology students from the United Kingdom. Participants had to fill in the Memory Beliefs Questionnaire (MBQ; Ost et al., 2017) containing 11 memory statements that have broad scientific consensus [e.g., “It is possible for an individual to develop false memories for a non-traumatic event” (true statement)]. Chartered Clinical Psychologists scored significantly more accurately on the memory statements than the hypnotherapists and the undergraduate psychology students. Among the hypnotherapists the authors found evidence for incorrect beliefs, such as the belief that memories from the first year of life can be retrieved accurately in adulthood. Some erroneous beliefs about memory appear to exist even among well-trained and experienced clinical psychologists. For example, 75% (*n* = 253) of the entire sample “strongly agreed” with the belief that “The mind is capable of unconsciously blocking out memories of traumatic events.” The findings of Ost et al. (2017) parallel the results of Patihis et al. (2013) who found that a large percentage of alternative therapists (including hypnotherapists) strongly agreed with false statements about memory (for example, 82% of hypnotherapists (*n* = 50) agreed that repressed and recovered memories can surface in therapy).

The above-mentioned studies indicate that different professional groups demonstrate a lack of accurate knowledge about memory in areas such as recovered memories. We searched the PsychInfo database using combinations of the following keywords: child protection workers, (memory) beliefs, (memory) knowledge and child interviewing, which yielded no results. To our knowledge, this is the first study of its kind conducted with child protection workers.

### The Present Study

The current study investigates memory beliefs in professionals from 26 Safe Home organizations in The Netherlands: social workers, behavioral scientists, and medical doctors. We also asked these professionals which child investigative interviewing method they are currently using and whether there is consensus within their organization about its application. Based on the literature described earlier, several hypotheses were formulated. First, we expect professionals to show inaccurate knowledge regarding memory functioning. Second, we expected a lack of use of child interviewing methods that have empirical support across all Safe Home organizations. Finally, we hypothesized that there also would be little consensus on child interviewing methods within each separate Safe Home organization.

## Materials and Methods

### Participants

Professionals (*N* = 154) from 26 Safe Home organizations in The Netherlands completed an online survey via Qualtrics. Our sample consisted of social workers (*n* = 100), behavioral scientists (*n* = 43) and medical doctors (*n* = 11).

Social workers who work at Safe Home have an educational background in social welfare or a Bachelor-level psychology degree. At Safe Home, their task is to investigate reports of alleged child abuse and domestic violence. Behavioral scientists are Master-level psychologists. Behavioral scientists supervise the social workers during their investigations. Also, behavioral scientists provide support during interviews with parents, informants, and victims. To be eligible to work as a behavioral scientist at Safe Home, licensing by the youth quality register (in Dutch: *Stichting Kwaliteits Register Jeugd*) is mandatory. Although social workers also need to be licensed, the criteria are different and educational background is less important. Medical doctors perform medical examinations in cases of child abuse reports, if deemed necessary. Medical doctors are required to have completed specialized training in investigating child abuse cases.

### Materials

In line with previous studies, we provided professionals with memory statements, selected or slightly adapted (e.g., “Memory is not influenced by suggestion” was adapted to “Memories cannot be influenced by suggestion”) from studies by Patihis et al. (2013) and Ost et al. (2017). We provided professionals with nine statements about memory and added one statement on the nature of questions during child investigative interviews. We selected these statements because they cover important topics for professionals working with child abuse cases (e.g., statements on suggestion and memory) and having wrong beliefs could have implications for their (field) work (e.g., suggestive interviewing techniques). The selected statements have been used in several earlier studies (e.g., Patihis et al., 2013; Ost et al., 2017; Houben et al., 2019). The statements were translated into Dutch by three independent individuals: one Master student in forensic psychology, one Ph.D. student, and a professor of Forensic Psychology chose the translation that fit best.

Participants had the option to either agree or disagree with the 10 statements. All participants viewed the questions and statements in the same order. We did not provide an intermediate option (*I don’t know* or *Neither agree nor disagree*), to prevent participants from answering neutrally. We asked participants which method(s) their Safe Home organization used for conducting child investigative interviews. This was a multiple choice question (four answer options) with an open comment area, in case the method the participant used was not listed among the four. Furthermore, we asked if consensus existed within their organization on applying a certain child interviewing method. We also asked whether they made use of additional tools (“props”) during their child interviews, such as anatomically correct dolls or drawings. This was also a multiple choice question with an open comment section. Finally, we asked participants whether they used a different interviewing method in cases of alleged child sexual abuse compared to other types of child abuse (and if so, which method they employed). The statements and response alternatives for memory knowledge are shown in Table 2, the answer choices consistent with current memory science are marked with an asterisk.

**TABLE 2:** Memory statements – the response alternative believed to be the most correct according to current memory science is indicated by an asterisk.

Statements	Response alternatives
1. Our memories are permanently stored in our brain, even though we can’t retrieve all of it.	Agree – Disagree*
2. Early memories, from the first year of life, are accurately stored and retrievable.	Agree – Disagree*
3. Repressed memories can be retrieved accurately by certain therapeutic techniques.	Agree – Disagree*
4. The more emotion with which a memory is reported, the more likely it is to be accurate.	Agree – Disagree*
5. Traumatic memories are often repressed because of their painful content.	Agree – Disagree*
6. It is possible for an individual to develop false memories for non-traumatic events.	Agree* – Disagree
7. Vivid memories are more likely to be accurate than vague memories.	Agree – Disagree*
8. A negative memory for a childhood event is indicative of a traumatic childhood.	Agree – Disagree*
9. Memories cannot be influenced by suggestion.	Agree – Disagree*
10. During a child forensic interview, directive questions should only be used when open questions have been exhausted.	Agree* – Disagree

**Statements reproduced/adapted from Patihis et al. (2013) and Ost et al. (2017).**

### Procedure

A link to the survey in Qualtrics was distributed by e-mail. We e-mailed all 72 behavioral scientists from the 26 Safe Home organizations in The Netherlands and used the snowball sampling method, asking them to distribute the survey among the other professionals (social workers, medical doctors or behavioral scientists) at their organization. A reminder was sent 2 weeks after the first e-mail. For the medical doctors, we made a request to the national forum (an online communication platform) for physicians at Safe Home. This request contained the same information as the e-mails sent to behavioral scientists as well as the link to the Qualtrics survey. No reminder was sent. The survey could be completed anonymously with the option of leaving an e-mail address to participate in a raffle for a 15 euro gift voucher. A completed questionnaire was obtained from 158 professionals. These professionals consisted of 43 behavioral scientists (27% of the total sample), 100 social workers (63%), and 11 medical doctors (7%) Approval for this study was received from the Ethical Review Committee of the Faculty of Psychology and Neuroscience at Maastricht University (ERCPN- 181_01_07_2017). Data from the current study are accessible via the Open Science Framework: https://osf.io/rjnz7/.

## Results

### Memory Beliefs

Table 3 shows the percentage of science-consistent answers to the memory statements. This is shown for the total sample and for the behavioral scientists, medical doctors and social workers, separately. The highest percentage of erroneous beliefs, that is beliefs that run counter to current memory research, were observed for statements regarding repressed and recovered memories. Seventy-four percent (*n* = 117) of the total sample agreed with the statement “Repressed memories can be retrieved accurately by certain therapeutic techniques.” The statement “Traumatic memories are often repressed because of their painful content” received 84% (*n* = 133) endorsement. Also, 64% (*n* = 101) of the total sample disagreed with the last statement “During a child forensic interview, directive questions should only be used when open questions have been exhausted.” High percentages of answers in line with current memory research were found for statement 6: “It is possible for an individual to develop false memories for non-traumatic events” (94.9%; *n* = 150) and statement 9: “Memories cannot be influenced by suggestion” (95%; *n* = 150).

**TABLE 3:** Percentage of behavioral scientists, medical doctors, social workers who responded correctly to10 statements about memory.

Statements	Behavioral scientists (*n* = 43)	Medical doctor (*n* = 11)	Social workers (*n* = 100)	Total (*N* = 154)
1. Our memories are permanently stored in our brain, even though we can’t retrieve all of it.	44 (*n* = 19)	73 (*n* = 8)	30 (*n* = 30)	37 (*n* = 57)
2. Early memories, from the first year of life, are accurately stored and retrievable.	93 (*n* = 40)	100 (*n* = 11)	89 (*n* = 89)	91 (*n* = 140)
3. Repressed memories can be retrieved accurately by certain therapeutic techniques.	23 (*n* = 10)	46 (*n* = 5)	24 (*n* = 24)	25 (*n* = 39)
4. The more emotion with which a memory is reported, the more likely it is to be accurate.	98 (*n* = 42)	82 (*n* = 9)	90 (*n* = 90)	92 (*n* = 141)
5. Traumatic memories are often repressed because of their painful content.	33 (*n* = 14)	9 (*n* = 1)	10 (*n* = 10)	16 (*n* = 25)
6. It is possible for an individual to develop false memories for non-traumatic events.	95 (*n* = 41)	100 (*n* = 11)	92 (*n* = 92)	94 (*n* = 144)
7. Vivid memories are more likely to be accurate than vague memories.	65 (*n* = 28)	73 (*n* = 8)	57 (*n* = 57)	60 (n = 93)
8. A negative memory for a childhood event is indicative of a traumatic childhood.	88 (*n* = 38)	73 (*n* = 8)	85 (*n* = 85)	85 (*n* = 131)
9. Memories cannot be influenced by suggestion.	98 (*n* = 42)	100 (*n* = 11)	92 (*n* = 92)	94 (*n* = 145)
10. During a child forensic interview, directive questions should only be used when open questions have been exhausted.	33 (*n* = 14)	64 (*n* = 7)	33 (*n* = 33)	35 (*n* = 54)

**TABLE 4:** Chi square analysis of behavioral scientists vs. social workers who responded inaccurately (%) on 10 statements about memory.

Statement	Behavioral scientists (*n* = 43)	Social workers(*n* = 100)	*χ^2^*	*p*-value (two-tailed)
1. Our memories are permanently stored in our brain, even though we can’t retrieve all of it.	24 (56)	70 (70)	2.69	0.101
2. Early memories, from the first year of life, are accurately stored and retrievable.	3 (7)	11 (11)	0.551	0.458
3. Repressed memories can be retrieved accurately by certain therapeutic techniques.	33 (77)	75 (75)	0.02	0.899
4. The more emotion with which a memory is reported, the more likely it is to be accurate.	1 (2)	9 (9)	2.10	0.148
5. Traumatic memories are often repressed because of their painful content.	29 (67)	88 (88)	10.57	**0.001**
6. It is possible for an individual to develop false memories for non-traumatic events.	2 (5)	6 (6)	0.12	0.728
7. Vivid memories are more likely to be accurate than vague memories.	15 (35)	41 (41)	0.603	0.437
8. A negative memory for a childhood event is indicative of a traumatic childhood.	5 (12)	13 (13)	0.072	0.789
9. Memories cannot be influenced by suggestion.	1 (2)	6 (6)	0.913	0.339
10. During a child forensic interview, directive questions should only be used when open questions have been exhausted.	29 (67)	65 (65)	0.017	0.897

*The bold value means the p value was considered significant.*

We also compared answers of the different professional groups on the memory statements by means of Chi Square analyses (Pearson χ^2^-test). Table 3 provides a comparison of Group 1 (behavioral scientists) and Group 2 (social workers). In The Netherlands, social workers do not have a Master’s degree, as opposed to behavioral scientists. Group 1 had more accurate knowledge on memory than Group 2. Specifically, there was a statistically significant difference (*χ*^2^ = 10.57, *p* = 0.001, *Cramer’s V* = 0.274) for the statement “Traumatic memories are often repressed because of their painful content”.

Additionally, Table 5 shows a bivariate correlational analysis of the 10 memory statements, which was added to investigate whether the questionnaire items are correlated with each other. As can be seen from the log odds ratios, not all items are positively related to each other. This finding was not expected (nine statements are supposedly related to each other, because they cover the topic of memory beliefs; only item 10 covered child interviewing).

**TABLE 5:** Log odds ratios for the bivariate comparisons of the 10 statements about memory.

	Item 1	Item 2	Item 3	Item 4	Item 5	Item 6	Item 7	Item 8	Item 9
Item 2	4.58								
Item 3	0.72	4.01							
Item 4	–0.26	–2.74	–0.54						
Item 5	0.73	–0.42	0.15	0.11					
Item 6	0.57	0.47	–0.60	0.47	0.23				
Item 7	0.84	0.14	–0.02	0.46	0.39	0.44			
Item 8	0.44	1.09	0.10	0.65	0.13	–2.60	1.04		
Item 9	–0.03	0.47	0.84	2.62	–1.24	–1.54	–0.68	–2.60	
Item 10	–0.10	–0.95	0.09	–1.28	–0.20	0.51	0.15	–0.34	–1.14

### Use of Interviewing Methods and Additional Tools

Our study shows that more than four out of 10 professionals (44.2%; *n* = 68) indicated they use the so-called Three Houses method from “Signs of Safety” (Turnell and Edwards, 1997) for interviewing children. Twenty-three percent (*n* = 35) of all professionals indicated they do not use a standardized method for conducting child investigative interviews. Twenty-eight percent (*n* = 43) responded they used another method than the options posed (Three Houses Method, self-developed questionnaire or no standardized method). In the open comment section, 21.5% (*n* = 34) of these professionals indicated they used a variety of interviewing methods at their organization, dependent on the child’s age and the nature and/or complexity of the reported child abuse. Furthermore, 86.4% (*n* = 133) answered that there are differences between professionals in the use of interviewing method(s) at their Safe Home organization, reflecting a lack of consensus and/or standardization.

Furthermore, about one in five professionals reported they used human figure drawings (18.9%; *n* = 29) or Duplo dolls (19.5%, *n* = 30) during child investigative interviews. Interviewing with Duplo dolls, which are similar to Playmobil dolls, was developed by Dutch therapist Marleen Diekmann Schoenmaker, who used Duplo dolls during her work with child victims of war in foreign countries (Diekmann Schoenmaker and Van der Veer, 2003). Because she did not speak the children’s mother language, Diekmann used Duplo dolls to facilitate communication with the children. Furthermore, 31.2% (*n* = 48) of the professionals reported using additional tools other than those listed in the web survey. In the open comment section, over 28 different tools were mentioned, such as smiley lists (a set of smileys that depict different emotions children can use to indicate how they feel), socio cards (cards with pictures of people or objects children can use to indicate which people or objects play an important role in their life), family compositions and the child’s own toys. Furthermore, 22.8% (*n* = 36) of the professionals indicated using multiple tools.

Forty-five percent of the professionals indicated they use a different interview method in cases of alleged child sexual abuse compared to other types of child abuse. Methods mentioned were: psycho-sexual screening (17%; a diagnostic tool to investigate the psychological and sexual development of the child) and a specific examination method for sexual abuse (9%). The latter method is intended to investigate “vague” signs of possible statutory sexual offending. Training in the examination method is offered by several private institutes in the Netherlands (e.g., Landelijk Opleidingscentrum Kindermishandeling; LOCK). An example of a “vague” sign is a child who says “I have secrets with my Dad and I am not allowed to tell.” Examination methods are designed to interview children in case there is not enough evidence for the police to start an investigation. In the open comment section, several social workers indicated that interviews in cases of alleged sexual abuse are only conducted by behavioral scientists or medical doctors at their Safe Home organization.

## Discussion

Consistent with our expectations, many professionals endorsed beliefs that are not in line with current scientific research on memory, especially controversial beliefs on the existence of repressed and recovered memories (see also Otgaar et al., 2019). Other memory beliefs of the professionals were largely in keeping with scientific consensus. Most professionals held accurate beliefs regarding the formation of false memories and the influence of suggestion on memory. However, the majority of professionals disagreed with the fact that directive questions should only be used in a child investigative interview when open prompt questions have been exhausted. This runs counter to current scientific literature, which emphasizes the importance of open prompts (also called “invitations”) to encourage children to provide a narrative of what they have experienced (Lamb et al., 2007). Since bivariate comparisons of the 10 memory statements showed that although expected, not all items were positively correlated to each other. Future research should therefore include more detailed analysis of underlying traits to explain these correlations.

We found that for questions on how memory works (e.g., “Our memories are permanently stored in our brain, even though we can’t retrieve all of it”), behavioral scientists responded more in line with memory science than social workers. Statistically significant differences were found for one of the 10 statements. This finding is in line with previous research that compared different professional groups on memory beliefs. For example, Patihis et al. (2013) found that psychology researchers were more accurate about the non-existence of repressed memories and agreed more often that memory can be unreliable compared to some psychology practitioner groups. Furthermore, in a recent study, Akhtar et al. (2018) found that police officers and the general public endorsed more erroneous memory beliefs, as opposed to memory experts who endorsed more scientifically supported memory beliefs.

Our results suggest that for some, but not all memory beliefs, there is a gap between beliefs held by professionals working at Safe Home in the Netherlands and the current scientific literature on memory. In line with earlier studies on memory beliefs (e.g., Patihis et al., 2013; Melinder and Magnussen, 2015; Ost et al., 2017) many professionals endorsed beliefs regarding repressed and recovered memories. The fact that the majority of our sample agreed that repressed memories can be retrieved accurately by certain therapeutic techniques raises concern. Empirical support for repression in adults who were abused as children is lacking (Corelli et al., 1997; Piper et al., 2008). One potential concern is that beliefs in repressed memories of child (sexual) abuse could bias professionals at Safe Home during the investigative process, affecting their interpretation of children’s behaviors or statements, and affecting their choice of questions during interviews with children.

As for child investigative interviewing, the majority of professionals disagreed with the statement “During a child forensic interview, directive questions should only be used when open questions have been exhausted.” Valuable and accurate information can be obtained by using open prompts (e.g., “Tell me what happened;” Lamb et al., 2018). Directive questions are focused prompts which, according to several empirical studies, actually elicit less relevant information from alleged child abuse victims than open prompts (Cyr and Lamb, 2009; Lamb et al., 2009). The fact that only a minority of the responding professionals were aware of this, raises concern that they will use directive questions too early in their child investigative interviews. Such early directive questions could in some cases become suggestive or leading.

As hypothesized, we found that interviewing methods that received empirical support are not being used in the majority of interviews with alleged victims of child abuse at Safe Home. The method reported most frequently (44.3%; *n* = 70) was the Three Houses method, which to our knowledge, lacks scientific support as a child investigative interviewing method. The Three Houses method is part of “Signs of Safety” (Turnell and Edwards, 1997). Signs of Safety was developed in Australia as a method for developing a constructive relationship between child protection workers and family members of (substantiated) abused children that re under child protection authority. Its purpose is to bring the child’s voice into the supervision and monitoring by child protective services. The child is instructed to draw three houses according to different themes: the house of worries (danger), the house of good things (safety) and the house of dreams (change). How the houses are drawn depends on the child’s characteristics (e.g., age, cognitive abilities, creativity). There are no predefined rules as to how every house should be drawn. Typically, the interviewer asks the child to draw and tell everything that is well and fine at home (safety), things that are not well at home (danger) and which things the child wold like to be improved (change). Previous research on the Three Houses method has focused primarily on the reported experiences of professionals, parents, and children with the method (for an overview, see Wheeler and Hogg, 2012). Research has shown that the Three Houses method stimulates open communication with families and it provides abusive parents more insight into the experiences of their children (Westbrook, 2006). The Three Houses method is, however, not designed to assist in fact-finding in cases of child abuse allegations, nor does it have supportive evidence that it helps optimize the retrieval of accurate and detailed memories. On the contrary, at face value, the instruction to draw a “dream house” could obviously elicit fantasies instead of memories.

Furthermore, 21% of the professionals reported that they did not use a standardized method for conducting child investigative interviews, 22% reported using multiple methods, and 87% indicated different professionals apply different methods at their Safe Home organization. This lack of a standardized child forensic interviewing method concurs with recent research among child protection professionals in the USA (Rivard and Compo, 2017). Rivard and Compo (2017) created an online survey which was distributed among Child Advocacy Centres and other child sexual abuse investigative agencies throughout the United States. This study also showed a diversity of interviewing protocols across agencies and professionals were trained in different methods. In most cases, Safe Home professionals interview children before the police become involved in a case, and the threshold for criminal investigation is not reached in every reported case. However, if such initial interviews are done incorrectly this could contaminate children’s memory, with obvious repercussions if the case is subsequently referred for further police investigation. Because child interviewing methods which have received empirical support are lacking, current practices raise doubts regarding the quality of statements collected from children by Safe Home.

Moreover, professionals indicated that additional tools, such as human figure drawings, anatomically correct dolls and Duplo dolls, are used during child investigative interviews. The use of such tools is highly controversial. To our knowledge, the use of Duplo dolls for child abuse investigative purposes has not been empirically investigated. Besides, several studies have demonstrated the harmful effects of anatomically correct dolls on children’s memory (e.g., Bruck et al., 2000) and the suggestive nature of these dolls can lead to false reports about sexual abuse. For example, Bruck et al. (2000) found that under certain conditions, the use of dolls increased incorrect reports of inappropriate behavior, such as touching body parts that were not actually touched. Also, studies have found that use of human figure drawings decreased the accuracy of children’s reports of events (Brown et al., 2007; Otgaar et al., 2012).

### Limitations

Before we draw our final conclusions, a number of limitations of the present study should be acknowledged. The current data were gathered by means of a snowball method: behavioral scientists working at Safe Home were asked to distribute the online survey among colleagues at their organization. This snowball method may have resulted in selection bias, in that especially professionals interested in memory and child investigative interviewing filled in the questionnaire. As a consequence of the snowball method we also do not know the exact response rate, because it is unclear how many colleagues of the behavioral scientists received the survey. Furthermore, the sample size of the medical doctors (*n* = 11) was quite small compared to the behavioral scientists and social workers. This could be due to the fact that for technical reasons no reminder e-mails could be sent to the medical doctors. In addition, our survey data on child investigative interviewing at Safe Home represent professionals’ self-report about their child interviewing practice. We do not know to what extent these data reflect actual practice. More specifically, these findings do not inform us about the frequency with which certain interview methods and/or additional tools are used. However, the finding that most surveyed professionals reported using non-scientifically supported child interviewing methods, is highly relevant.

## Conclusion

Taken together, our study suggests a need for training of Safe Home professionals in both memory functioning and the practice of evidence-based child investigative interviewing. In line with previous research (Patihis et al., 2013; Ost et al., 2017; Patihis and Pendergrast, 2019), incorrect knowledge, on issues such as repressed memories for trauma, are common among professionals working in child protection. Furthermore, professionals do not use a child interviewing method which has received empirical support and they use additional tools, such as human figure drawings, that increase the risk of false memory reports (Otgaar et al., 2012). Because Safe Home is the Dutch “first responder” organization that deals with reports of alleged child abuse by both citizens and professionals, its professionals should be appropriately trained in the relevant knowledge and skills. Most of the required knowledge and skills are not taught in these professionals’ primary educational programs (e.g., social work, child psychology).

We recommend implementing evidence-based child interviewing methods, for example, the National Institute of Child Health and Development (NICHD) interview protocol, developed by Lamb et al. (2007). This interview-protocol was developed to obtain reliable and detailed accounts from children by the use of open prompts (“Tell me what happened”) that facilitate free recall from episodic memory (Olafson, 2012; La Rooy et al., 2015). Only when these open prompts do not elicit relevant material any longer, the interviewer can use directive questions (e.g., “Where did he touch you?”- when the child has already disclosed being touched) or option-posing questions, where the child can choose between two different answers (e.g., “Did he touch you above or beneath your clothes?”). The protocol consists of different phases, including rapport building, explaining ground rules (for example, what it means to tell the truth), training in episodic memory, substantive phase (asking about the alleged events), and closing with a neutral topic. Interviewers trained in the NICHD protocol tend to use more open prompts and fewer suggestive questions than they did before training (e.g., Yi et al., 2016).

Our findings are worrisome because workers at Safe Home are the first line of professionals who interview children about an alleged experience of abuse. Incorrect memory beliefs and deficient child interviewing methods could result in false positives (concluding a child was abused, while actually the child was not) or false negatives (concluding the child was not abused, when the child in fact was abused). Hence, it is vital that professionals at Safe Home adopt empirically-based methods for interviewing children, and that they are educated on the science of memory relevant to applied settings.

## Data Availability Statement

The datasets generated for this study can be found in online repositories. The names of the repository/repositories and accession number(s) can be found at: https://osf.io/rjnz7/.

## Ethics Statement

The studies involving human participants were reviewed and approved by the Ethical Review Committee Psychology and Neurocience. The patients/participants provided their written informed consent to participate in this study.

## Author Contributions

All authors have contributed to the manuscript and provided approval for the publication.

## Conflict of Interest

The authors declare that the research was conducted in the absence of any commercial or financial relationships that could be construed as a potential conflict of interest.

## References

[B1] Akhtar S. Justice L. V. Knott L. Kibowski F. Conway M. A. (2018). The ‘common sense’ memory belief system and its implications. *Int. J. Evid. Proof* 22 289–304. 10.1177/1365712718784045

[B2] Brown D. A. Pipe M. Lewis C. Lamb M. E. Orbach Y. (2007). Supportive or suggestive: do human figure drawings help 5- to 7-year-old children to report touch? *J. Consult. Clin. Psychol.* 75 33–42. 10.1037/0022-006X.75.1.33 17295561

[B3] Bruck M. Ceci S. J. Francoeur E. (2000). Children’s use of anatomically detailed dolls to report genital touching in a medical examination: development and gender comparisons. *J. Exp. Psychol. Appl.* 6 74–83. 10.1037//0278-7393.6.1.74 10937313

[B4] Ceci S. J. Bruck M. (1993). Suggestibility of the child witness: a historical review and synthesis. *Psychol. Bull.* 113 403–439. 10.1037/0033-2909.113.3.403 8316609

[B5] Ceci S. J. Gilstrap L. Fitneva S. (2002). *Children’s Testimony. Child and Adolescent Psychiatry.* Oxford: Blackwell Science, 117–127.

[B6] Central Bureau of Statistics (2017). *Veilig Thuis Eerste Halfjaar 2017: Nog Steeds Geen Vergelijkbare Cijfers. [Safe Home, First Sixth Months of 2017: Still No Comparable Numbers*.]. Thapathali: Central Bureau of Statistics.

[B7] Child protection system in England (2019). Available online at: https://learning.nspcc.org.uk/child-protection-system/england/

[B8] Corelli T. B. Hoag M. J. Howell R. J. (1997). Memory, repression, and sexual abuse: forensic implications for the mental health professional. *J. Am. Acad. Psychiatry Law* 25 31–47. 9148881

[B9] Cross T. P. Hershkowitz I. (2017). Psychology and child protection: promoting widespread improvement in practice. *Psychol. Public Policy Law* 23 503–518. 10.1037/law0000141

[B10] Cyr M. Lamb E. M. (2009). Assessing the effectiveness of the NICHD investigative interview protocol when interviewing French-speaking alleged victims of child sexual abuse in Quebec. *Child Abuse Negl.* 33, 257–268. 10.1016/j.chiabu.2008.04.002 19481261

[B11] Dammeyer M. D. Nightingale N. N. McCoy M. L. (1997). Repressed memory and other controversial origins of sexual abuse allegations: beliefs among psychologists and clinical social workers. *Child Maltreat.* 2 252–263. 10.1177/1077559597002003007

[B12] Dekker S. (2018). Verbetering Feitenonderzoek in de Jeugdbeschermingsketen. [Improvement of Evidence-Based Investigation in the Youth Care System.] Available online at: https://www.tweedekamer.nl/kamerstukken/brieven_regering/detail?id=2018Z 0662anddid=2018D32523

[B13] Diekmann Schoenmaker M. Van der Veer G. (2003). An extra language in counseling and training. *Intervention* 1 36–39.

[B14] Euser E. M. van IJzendoorn M. H. Prinzie P. Bakermans-Kranenburg M. J. (2010). Prevalence of child maltreatment in the Netherlands. *Child Maltreat.* 15 5–17. 10.1177/1077559509345904 19729577

[B15] Garven S. Wood J. M. Malpass R. S. Shaw J. S. III (1998). More than suggestion: the effect of interviewing techniques from the McMartin Preschool case. *J. Appl. Psychol.* 83 347–359. 9648524 10.1037/0021-9010.83.3.347

[B16] Goodman G. S. Melinder A. (2007). Child witness research and forensic interviews of young children: a review. *Legal Criminol. Psychol.* 12 1–19. 10.1348/135532506X156620 30467716

[B17] Gore-Felton C. Koopman C. Thoresen C. Arnow B. Bridges E. Spiegel D. (2000). Psychologists’ beliefs and clinical characteristics: judging the veracity of childhood sexual abuse memories. *Prof. Psychol. Res. Pract.* 31 372–377. 10.1037/0735-7028.31.4.372

[B18] Houben S. T. L. Otgaar H. Roelofs J. Wessel I. Patihis L. Merckelbach H. (2019). Eye movement desensitization and reprocessing (EMDR) practitioners’ beliefs about memory. *Psychol. Conscious. Theory Res. Pract.* 10.1037/cns0000211 [Epub ahead of print].

[B19] Huijer J. (2014). Fact finding in the youth care system. A legal perspective. *Nederlands Juristenblad* 13 834–837.

[B20] Inspectie Jeugdzorg (2014). *Annual Report.* Available online at: www.inspectiejeugdzorg.nl

[B21] Kinderombudsman (2013). *Is de Zorg Gegrond? Analyse van het Feitenonderzoek aan de Basis van Ingrijpende Jeugdzorgbeslissingen. [Is the Concern Well-Founded? Analysis of Evidence-Based Research in Far-Reaching Youth Care Decisions].* Available online at: http://www.dekinderombudsman.nl/ul/cms/fckuploaded/2013.KOM008Isdezorggegr nd.pdf

[B22] Kwakman E. (2017). *Handreiking Samenwerken bij Strafbare Kindermishandeling. [Guide to Collaboration in Criminal Child Abuse Cases].* Available online at: https://www.rijksoverheid.nl/documenten/publicaties/2017/11/09/handreiking samenwerken-bij-strafbare-kindermishandeling

[B23] La Rooy D. Brubacher S. P. Aromaki-Stratos A. Cyr M. Hershkowitz I. (2015). The NICHD protocol: a review of an internationally-used evidence-based tool for training child forensic interviewers. *J. Criminol. Res. Policy Pract.* 1 76–89. 10.1108/JCRPP-01-2015-0001

[B24] Lamb M. E. Brown D. A. Hershkowitz I. Orbach Y. Esplin P. W. (2018). *Tell Me What Happened: Questioning Children About Abuse.* Chichester: Wiley.

[B25] Lamb M. E. La Rooy D. J. Malloy L. C. Katz C. (2011). *Children’s Testimony: A Handbook of Psychological Research and Forensic Practice.* Chichester: Wiley.

[B26] Lamb M. E. Orbach Y. Herkowitz I. Esplin P. W. Horowitz D. (2007). A structured forensic interview protocol improves the quality and informativeness of investigative interviews with children: a review of research using the NICHD investigative interview protocol. *Child Abuse Neglect* 31 1201–1231. 10.1016/j.chiabu.2007.03.021 18023872 PMC2180422

[B27] Lamb M. E. Orbach Y. Sternberg K. J. Aldridge J. Pearson S. Steward H. L. (2009). Use of a structured investigative protocol enhances the quality of investigative interviews with alleged victims of child sexual abuse in Britain. *Appl. Cogn. Psychol.* 23 449–467. 10.1002/acp.1489

[B28] Lindsay D. S. (1990). Misleading suggestions can impair eyewitnesses’ ability to remember event details. *J. Exp. Psychol. Learn. Mem. Cogn.* 16 1077–1083.

[B29] McElvaney R. (2015). Disclosure of child sexual abuse: delays, non-disclosure and partial disclosure. What the research tells us and implications for practice. *Child Abuse Rev.* 24 159–169. 10.1002/car.2280

[B30] Melinder A. (2002). “Children’s memory of a mildly stressful event – what help is helpful?,” in *Studies of the Mind: Proceedings of the First Norwegian Cypriot Meeting on Cognitive Psychology and Neuropsychology*, eds Korsnes M. S. Raftopoulos A. Demetriou A. (Nicosia: Cyprus University Press), 25–31.

[B31] Melinder A. Goodman G. S. Eilertsen D. E. Magnussen S. (2004). Beliefs about child witnesses: a survey of professionals. *Psychol. Crime Law* 10 347–365. 10.1080/10683160310001618717

[B32] Melinder A. Magnussen S. (2015). Psychologists and psychiatrists serving as expert witnesses in court: what do they know about eyewitness memory? *Psychol. Crime Law* 21 53–61. 10.1080/1068316X.2014.915324

[B33] Merckelbach H. Wessel I. (1998). Assumptions of students and psychotherapists about memory. *Psychol. Rep.* 82 763–770. 10.2466/pr0.1998.82.3.763 9676488

[B34] Olafson E. (2012). A call for field-relevant research about child forensic interviewing for child protection. *J. Child Sex. Abuse* 21 109–129. 10.1080/10538712.2012.642469 22339427

[B35] Ost J. Easton S. Hope L. French C. C. Wright D. B. (2017). Latent variables underlying memory beliefs of chartered clinical psychologists, hypnotherapists and undergraduate students. *Memory* 25 57–68. 10.1080/09658211.2015.1125927 26728198

[B36] Otgaar H. de Ruiter C. Howe M. L. Hoetmer L. van Reekum P. (2017). A case concerning children’s false memories of abuse: recommendations regarding expert witness work. *Psychiatry Psychol. Law* 24 365–378. 10.1080/13218719.2016.1230924 31983961 PMC6818307

[B37] Otgaar H. Horselenberg R. van Kampen R. Lalleman K. (2012). Clothed and unclothed human figure drawings lead to more correct and incorrect reports of touch in children. *Psychol. Crime Law* 18 641–653. 10.1080/1068316X.2010.532129

[B38] Otgaar H. Howe M. L. Merckelbach H. Muris P. (2018). Who is the better eyewitness? Sometimes children but at other times adults. *Curr. Dir. Psychol. Sci.* 27 378–385. 10.1177/0963721418770998 30369724 PMC6187487

[B39] Otgaar H. Howe M. L. Patihis L. Merckelbach H. Lynn S. J. Lilienfeld S. O. (2019). The return of the repressed: the persistent and problematic claims of long-forgotten trauma. *Perspect. Psychol. Sci.* 14 1072–1095. 10.1177/1745691619862306 31584864 PMC6826861

[B40] Patihis L. Ho L. Y. Tingen I. W. Lilienfeld S. O. Loftus E. F. (2013). Are the ‘Memory Wars’ over? A scientist-practitioner gap in beliefs about repressed memories. *Psychol. Sci.* 25 519–530. 10.1177/0956797613510718 24335599

[B41] Patihis L. Pendergrast M. H. (2019). Reports of recovered memories of abuse in therapy in a large age-representative U.S national sample: therapy type and decade comparisons. *Clin. Psychol. Sci.* 7 3–21. 10.1177/2167702618773315

[B42] Piper A. Lillevik L. Kritzer R. (2008). What’s wrong in believing in repression? A review for legal professionals. *Psychol. Public Policy Law* 3 223–242. 10.1037/a0014090

[B43] Rivard J. R. Compo N. S. (2017). Self-reported current practices in child forensic interviewing: training, tools and pre-interview preparation. *Behav. Sci. Law* 35 253–268. 10.1002/bsl.2290 28581153

[B44] Turnell A. Edwards S. (1997). Aspiring to partnership, the signs of safety approach to child protection. *Child Abuse Rev.* 6 179–190.

[B45] Westbrook S. (2006). *Utilizing the Signs of Safety Framework to Create Effective Relationships with Child Protection Service Recipients.* St. Paul, MN: MSW Clinical Research.

[B46] Wheeler J. Hogg V. (2012). “Signs of safety and the child protection movement,” in *Solution-Focused Brief Therapy. A Handbook of Evidence-Based Practice*, eds Franklin C. Trepper T. S. Gingerich W. McCollum E. E. (London, UK: Oxford University Press), 203–215.

[B47] Yi M. Jo E. Lamb M. E. (2016). Effects of the NICHD protocol training on child investigative interview quality in Korean police officers. *J. Police Crim. Psychol.* 31 155–163.

